# Supplementary material for: Effects of a three-week executive control training on adaptation to task difficulty and emotional interference
Source: PLoS One. 2022 Nov 22;17(11):e0276994. doi: 10.1371/journal.pone.0276994 (PMC9681094; doi:10.1371/journal.pone.0276994)
Supplement: S1 File — (DOCX) [file pone.0276994.s001.docx]

Supplement 1: IAPS pictures

The following neutral pictures were included: 1350,1450,1670, 2036, 2038, 2191, 2273, 2359, 2377, 2383, 2384, 2435, 2480, 2518, 2745.1, 2479, 2850, 2880, 2980, 5040, 5390, 5520, 5530, 5635, 5720, 5740, 6150, 7000, 7004, 7009, 7010, 7021, 7026, 7035, 7045, 7059, 7090, 7140, 7150, 7175, 7185, 7186, 7233, 7290, 7490, 7491, 7500, 7512.

The following negative pictures were included: 2053, 2301, 2345.1, 2375.1, 2456, 2811, 2981, 3019, 3051, 3195, 3212, 3213, 3220, 3500, 3500.1, 6021, 6231, 6250.1, 6300, 6311, 6312, 6350, 6370, 6560, 6570, 6821, 6838, 8330, 8485, 9000, 9041, 9140, 9180, 9253, 9419, 9423, 9560, 9561, 9600, 9610, 9611, 9630, 9902, 9904, 9908, 9909, 9910, 9911.

Supplement 2: OCD picture set

As the data was collect as part of a pilot study, assessing the training’s suitability for application in obsessive-compulsive disorder (OCD), OCD-specific pictures were presented on 1/3 of the trials in each block (i.e. in 96 trials). Pictures were extracted from the Maudsley Obsessive-Compulsive Stimuli Set (Mataix-Cols, Lawrence, Wooderson, Speckens, & Phillips, 2009) and the Berlin Obsessive-Compulsive Disorder Picture System (Simon, Kischkel, Spielberg, & Kathmann, 2012).

Pictures for the most common symptom dimensions were extracted: 1. Contamination/washing, 2. checking, 3. symmetry. Additionally, picture array for patients with multiple symptom dimensions were created with an equal proportion of sub-dimension pictures: 4. contamination and checking, 5. contamination and symmetry, 6. checking and symmetry, 7. washing, checking and symmetry. Each picture set contained 48 pictures.

In the main study with OCD patients, pictures sets will be assigned individually based on the patient’s predominant symptom dimension(s). In the present study, OCD picture set were randomly assigned to participants. The same picture set was presented at pre- and post-training. In the present sample eight partipants received stimulus set 1 (contamination/washing), seven received set 2 (checking), eight received set 3 (symmetry) and one received set 5 (contamination and symmetry).

Supplement 2: Proportion congruency effect on EEG data


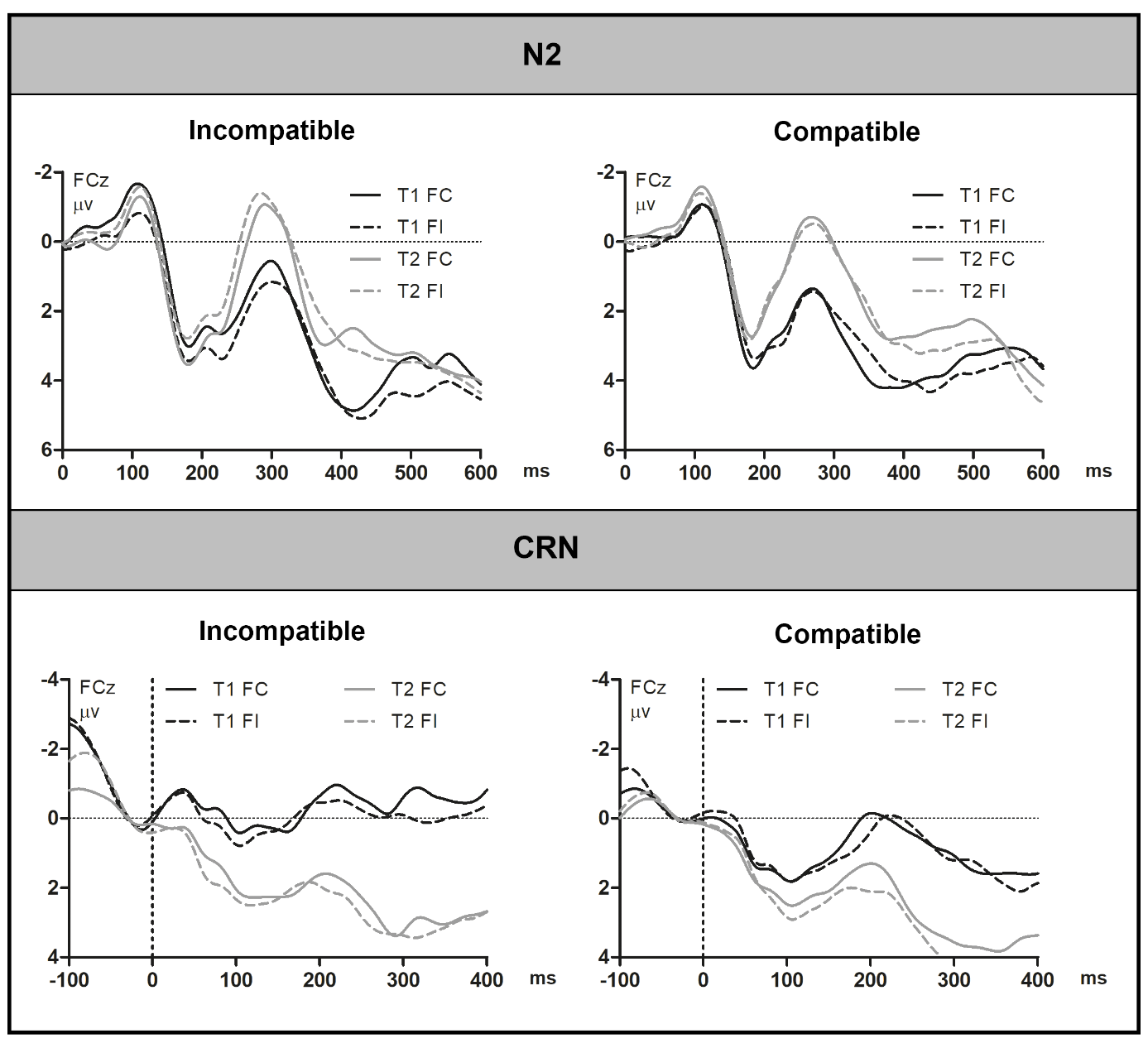


Figure S1: Grand Averages of the N2 (upper panel) and CRN (lower panel) in incompatible and compatible trials in the FC and FI condition. In order to reflect the proportion congruency effect independent of picture valence, ERPs for neutral and negative trials were averaged.

References

Mataix-Cols, D., Lawrence, N. S., Wooderson, S., Speckens, A., & Phillips, M. L. (2009). The Maudsley Obsessive–Compulsive Stimuli Set: Validation of a standardized paradigm for symptom-specific provocation in obsessive–compulsive disorder. *Psychiatry Research, 168*, 238-241. doi:<https://doi.org/10.1016/j.psychres.2008.05.007>

Simon, D., Kischkel, E., Spielberg, R., & Kathmann, N. (2012). A pilot study on the validity of using pictures and videos for individualized symptom provocation in obsessive-compulsive disorder. *Psychiatry Res, 198*, 81-88. doi:10.1016/j.psychres.2011.12.022

S0165-1781(11)00806-7 [pii]
